# Electride‐Induced Electronic Modulation of Ruthenium Catalyst for Highly Efficient Alkaline Hydrogen Evolution

**DOI:** 10.1002/advs.74939

**Published:** 2026-03-24

**Authors:** Zhiqi Wang, Jianan Su, Cheng Li, Yutong Gong, Hideo Hosono, Junjie Wang

**Affiliations:** ^1^ State Key Laboratory of Solidification Processing School of Materials Science and Engineering Northwestern Polytechnical University Xi'an Shaanxi P. R. China; ^2^ Xi'an Key Laboratory of Intelligent Design of Energy Catalytic Materials Northwestern Polytechnical University Xi'an Shaanxi P. R. China; ^3^ MDX Research Center for Element Strategy Institute of Integrated Research Institute of Science Tokyo Yokohama Japan

**Keywords:** electrides, electronic structure engineering, hydrogen evolution reaction, ruthenium electrocatalyst

## Abstract

Electrides, featuring localized electrons serving as anionic species, have attracted increasing attention for their catalytic potential. However, their application as supports for hydrogen evolution reaction (HER) electrocatalysts has remained elusive, primarily due to their high chemical reactivity under aqueous conditions. In this study, we report the rational design of a robust electride‐supported HER catalyst, Ruthenium (Ru)/Nd_2_ScSi_2_, which delivers outstanding performance with an overpotential of only 48 mV at −10 mA cm^−2^ in 1.0 m KOH, along with excellent long‐term stability. In‐depth mechanistic investigations, combining in situ spectroscopic analyses with density functional theory calculations, reveal that negatively charged Ru species anchored on the electride surface are pivotal in promoting HER activity. These anionic Ru sites facilitate water dissociation and modulate hydrogen adsorption by reshaping the electronic landscape and tuning the active sites. Our findings highlight the feasibility of leveraging the unique electronic characteristics of electrides to modulate supported metal catalysts, offering a new paradigm for the design of high‐efficiency HER electrocatalysts through electronic structure engineering.

## Graphical abstract

Electrides offer unique opportunities as catalyst supports for hydrogen evolution reactions. This study presents an electride‐supported Ru/Nd_2_ScSi_2_ catalyst exhibiting outstanding hydrogen evolution reaction performance and excellent stability. Anionic Ru species on the electride surface facilitate water dissociation and optimize hydrogen adsorption energy. This work opens a new avenue for designing efficient electrocatalysts via electronic structure engineering.

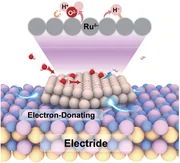

## 1 Introduction

Electrides are a unique class of materials in which localized electrons occupy interstitial sites and act as anions, resulting in unusual electronic structures and properties—most notably, low work functions and strong electron‐donating capabilities [1, 2]. A breakthrough in this field was the discovery of the first inorganic electride, [Ca_24_Al_28_O_64_]^4+^(e^−^)_4_ (C12A7:e^−^), by Hosono et al., which opened avenues for practical applications [3]. Since then, electrides have demonstrated promising potential in catalysis [4, 5, 6], electron emission [7], and superconductivity [8]. Their interstitial electrons and low work functions make them particularly effective in catalytic processes such as ammonia synthesis [4, 5, 9], CO_2_ reduction [10], and Suzuki coupling [11]. For instance, in ammonia synthesis, electrides facilitate N_2_ activation and reversible hydrogen storage, enhancing catalyst efficiency [12, 13, 14, 15]. Despite these advantages, the high chemical reactivity of electrides has traditionally limited their use in aqueous and electrochemical environments. Recent advances have led to the development of water‐stable electrides, such as Y_5_Si_3_ [16], LaTMSi (TM = Co, Fe, Ru) [9], TM/La‐Al‐N [17], and *A*_2_ *BC*_2_‐type compounds [18], enabling exploration of electrochemical catalytic properties.

Among electrochemical applications, the hydrogen evolution reaction (HER) is of particular importance due to hydrogen's role in clean energy systems [19, 20, 21, 22]. Alkaline water electrolysis, a long‐standing method for hydrogen production [23], faces a key challenge: sluggish HER kinetics, largely due to the energy barrier associated with water dissociation in the Volmer step (H_2_O + e^−^ → H^*^ + OH^−^) [24, 25]. As a result, Pt/C catalysts exhibit HER rates in alkaline media that are 2–3 orders of magnitude lower than in acid [26, 27]. Developing efficient and stable HER catalysts under alkaline conditions is thus a pressing need.

Ruthenium (Ru)‐based catalysts are promising alternatives to Pt due to their similar catalytic properties, faster water dissociation kinetics, and lower cost [28, 29, 30]. However, Ru tends to strongly bind hydrogen intermediates (H^*^), which hinders hydrogen desorption [31]. Therefore, strategies to tune H* binding energies are crucial for optimizing Ru‐based catalysts [32, 33]. One promising strategy involves constructing Ru with negatively charged states. While positively charged Ru can improve HER activity [34, 35], it is unstable under reductive conditions, often reverting to metallic Ru and losing activity. In contrast, negatively charged Ru species are more resistant to reduction, offering better long‐term performance. Yet, studies on such reduced‐valence Ru for HER are scarce.

Electrides, characterized by their intrinsically low work functions, arising from nuclear‐free anionic nature, can effectively donate electrons to supported metal species, thereby facilitating the formation of reduced and catalytically active metal sites [12, 14, 36]. For instance, in the LaRuSi electride, Si atoms act as the primary HER active sites due to their favorable hydrogen adsorption properties [37]. Similarly, selective Ce removal in CeRuSi electride modulates the Ru *d*‐band structure, resulting in enhanced catalytic performance [38]. Despite these promising features, the high Ru content and the limited accessibility to active sites, most of which are embedded within the bulk, pose significant challenges for practical applications. These examples highlight the promise of electrides not only as active HER catalysts but also as effective catalyst supports. However, a critical knowledge gap remains: the role of electrides as supports in Ru‐based HER systems is still poorly understood. This limitation largely arises from the scarcity of electrides that simultaneously possess a high density of anionic electrons and sufficient chemical stability. Addressing this gap calls for the rational design of electride supports that can stabilize low Ru loadings while maximizing the exposure of active sites, thereby unlocking their full catalytic potential.

In this study, we report the first supported electride HER catalyst based on Ru/Nd_2_ScSi_2_. We first confirm the intrinsic electride nature of Nd_2_ScSi_2_ via electronic structure analysis. Using chemical vapor deposition (CVD) of Ru, we synthesize Ru/Nd_2_ScSi_2_ and characterize its structure and electronic properties. The catalyst with 7.2 wt.% Ru exhibits an overpotential of just 48 mV at −10 mA cm^−2^ in 1 m KOH, comparable to commercial Pt catalysts. Moreover, its mass activity ranks among the highest for electride‐based systems. Mechanistic insights from in situ infrared spectroscopy and density functional theory (DFT) calculations reveal that the negatively charged Ru sites in Ru/Nd_2_ScSi_2_ significantly lower the energy barrier for water dissociation and optimize H^*^ binding, resulting in outstanding alkaline HER activity. These findings demonstrate the potential of electrides as versatile supports for Ru‐based electrocatalysts and provide a new framework for catalyst design through electronic structure engineering.

## 2 Results and Discussion

### 2.1 Theoretical Calculations on Nd_2_ScSi_2_

**FIGURE 1:**
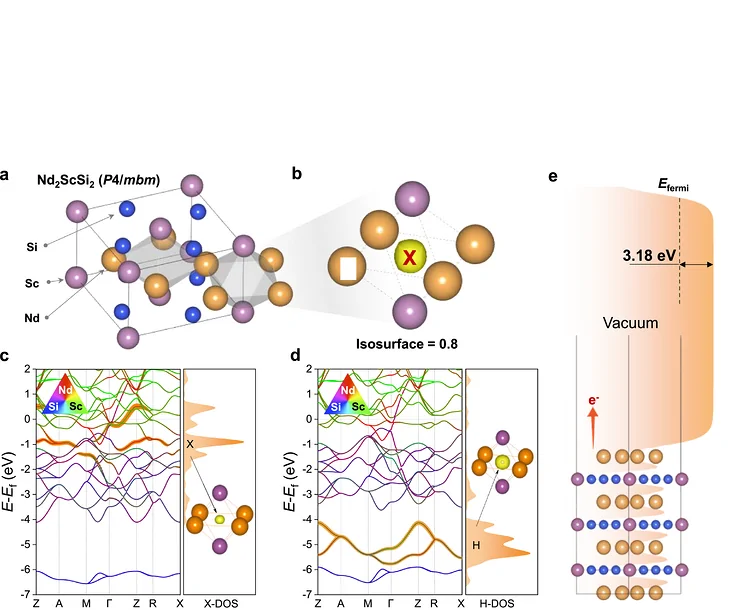
Crystal and electronic structures of Nd_2_ScSi_2_. (a) Crystal structure of Nd_2_ScSi_2_. (b) Electron Localization Function (ELF) map, highlighting highly localized interstitial electrons within octahedral voids. (c) Band structure and density of states (DOS) of Nd_2_ScSi_2_, with the orange‐highlighted bands indicating the contribution from interstitial electrons. (d) Band structure and DOS of Nd_2_ScSi_2_H, with orange‐shaded regions indicating the projected H‐derived electronic states. (e) Calculated work function of the Nd_2_ScSi_2_─Nd(001) surface.

Nd_2_ScSi_2_ is a newly identified electride that exhibits outstanding catalytic performance for ammonia synthesis under low‐pressure conditions [18]. Crystallizing in the *P*4/*mbm* space group, Nd_2_ScSi_2_ features an alternating layered arrangement of Nd and Sc─Si units along the (001) direction [18, 39]. As illustrated in Figure 1a, both Nd and Sc atoms contribute to an octahedral coordination environment, with Sc atoms occupying two vertices of each octahedron. First‐principles calculations of the electron localization function (ELF) reveal a high degree of interstitial electron localization within the voids of octahedra, as shown in Figure 1b. Notably, the ELF reaches a maximum value of 0.88 at the interstitial site located at fractional coordinates (0.5, 0.5, 0.5), indicating a strongly confined electron density characteristic of electride behavior.

Figure 1c displays the projected electronic band structure and corresponding density of states (DOS) for Nd_2_ScSi_2_. The interstitial electrons are mainly distributed within 2.0 eV below the Fermi level and exhibit band dispersion across it, indicative of zero‐dimensional electride characteristics. Upon hydrogen incorporation into the interstitial voids, the two bands near the Fermi level, which are associated with the two interstitial sites per unit cell, disappear, and two new bands emerge around −5 eV, attributed to hydrogen 1*s* orbitals, as shown in Figure 1d. This transformation reflects a strong hydrogen‐trapping tendency at the interstitial sites. The calculated H_2_ desorption energy from Nd_2_ScSi_2_H is 1.8 eV (Figure S1), comparable to that of the one‐dimensional electride Sr_5_P_3_ (2.02 eV) [40], suggesting favorable thermodynamics for H^−^ formation and subsequent H_2_ release.

Another important feature arising from the presence of interstitial electrons with a high energy level is the low work function. Figure 1e presents the electrostatic potential along the (001) surface, from which a work function of 3.18 eV is obtained. This relatively low value is attributed to the ease of interstitial electron excitation across the Fermi level, underscoring the strong electron‐donating capability of Nd_2_ScSi_2_, a critical attribute for catalytic applications such as ammonia synthesis.

### 2.2 Experimental Synthesis and Characterization of Nd_2_ScSi_2_

**FIGURE 2:**
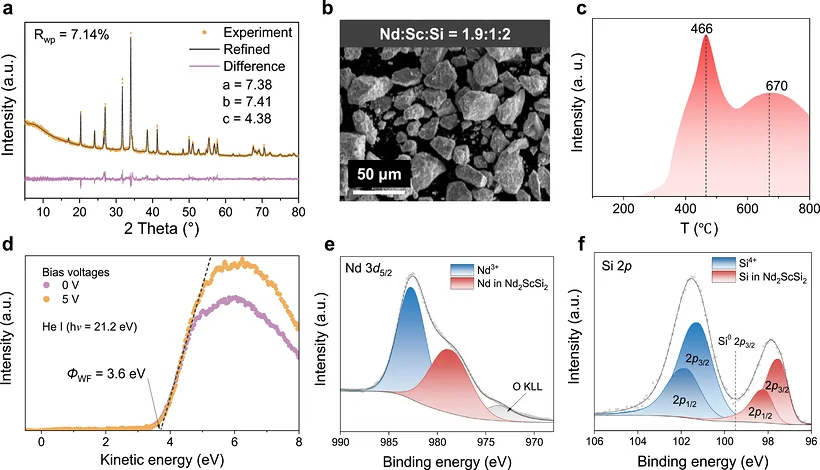
Structural and physicochemical characterization of the Nd_2_ScSi_2_. (a) Rietveld‐refined XRD pattern of Nd_2_ScSi_2_. (b) SEM image of Nd_2_ScSi_2_. (c) H_2_ temperature‐programmed desorption profile. (d) The measured work function of Nd_2_ScSi_2_ by ultraviolet photoelectron spectroscopy. The cut‐off energy of secondary electrons remains unchanged with changing sample bias voltage, confirming the reliability of the measured work function. (e, f) X‐ray photoelectron spectroscopy spectra of Nd and Si species on the Nd_2_ScSi_2_ surface.

The Nd_2_ScSi_2_ sample was synthesized using a solid‐state reaction method. As shown in Figure 2a, the X‐ray diffraction (XRD) pattern of the as‐prepared material yields a low *R*_wp_ value of 7.14%, demonstrating excellent agreement between the experimental data and the calculated diffraction profile. This confirms that the obtained sample crystallizes in the correct structure with negligible impurity phases. The scanning electron micrope (SEM) image in Figure 2b reveals that the Nd_2_ScSi_2_ support consists of particles with sizes on the order of tens of micrometers. Elemental composition determined by energy dispersive X‐ray spectroscopy (EDX) analysis gives an atomic ratio of Nd:Sc:Si = 1.9:1:2 (Figure 2b), which is consistent with the expected stoichiometry.

To evaluate the strong trapping ability of interstitial electrons for hydrogen, temperature‐programmed desorption (TPD) experiments were conducted (Figure 2c). Two desorption peaks at 466°C and 670°C correspond to the release of surface‐adsorbed hydrogen and bulk hydrogen, respectively. DFT calculation shows that hydrogen diffusion along the [001] direction in Nd_2_ScSi_2_ has a barrier of 0.97 eV (Figure S2), indicating that bulk hydrogen requires higher temperatures to overcome the migration barrier and reach the surface, consistent with the second desorption peak. Ultraviolet photoelectron spectroscopy (UPS) measurements revealed a work function of 3.6 eV for Nd_2_ScSi_2_ (Figure 2d). A lower work function corresponds to an enhanced electron‐donating capability, enabling the supported metal to adopt uncommon reduced states, which are beneficial for catalytic applications [12, 13, 41]. Considering the inevitable presence of a thin surface oxide layer, this value is in good agreement with the theoretically calculated work function of the (001) surface (3.18 eV).

Further structural characterization by transmission electron microscopy (TEM) is presented in Figure S3. Top‐view TEM images clearly show well‐resolved lattice fringes corresponding to the (300)/(030) and (120) planes, both oriented perpendicular to the [001] direction. The measured lattice spacings, ∼2.47 Å for (300)/(030) and ∼3.37 Å for (120), are in good agreement with the values derived from the XRD analysis (2.47 Å and 3.35 Å, respectively). Together with the work function measurements described above, these observations indicate that Nd_2_ScSi_2_ crystals preferentially expose the (001) surface. Importantly, this facet is predicted to host a high density of interstitial electrons and catalytically active sites (Figure 1e), which are essential for facilitating electron transfer and enhancing catalytic activity. Thus, the preferential (001) surface orientation is expected to play a decisive role in governing the electronic structure and catalytic performance of Nd_2_ScSi_2_ electride.

To gain a deeper understanding of the electronic structure of Nd_2_ScSi_2_, X‐ray photoelectron spectroscopy (XPS) measurements were carried out, with all binding energies calibrated to the C 1*s* peak at 284.8 eV (Figure S4). As shown in Figure 2e, the Nd 3*d*_5/2_ binding energy of Nd_2_ScSi_2_ is lower than that of reference Nd^3+^, and a similar downshift is also observed for Sc (Figure S5). This simultaneous shift can be attributed to the presence of interstitial electrons confined within the Nd‐Sc octahedral framework. The projected band structure (Figure 1c) further confirms the hybridization between these interstitial electrons and Nd/Sc orbitals, which effectively weakens the core‐level electron binding of Nd and Sc, thereby leading to the reduced binding energies observed in XPS.

For Si, the XPS spectrum (Figure 2f) displays a 2*p*_3/2_ binding energy of 97.6 eV, which is significantly lower than that of elemental Si^0^ (99.0 eV) [42]. This pronounced negative energy shift demonstrates that Si participates in the anionic framework. Kadono and co‐workers reported that Si in Mg_2_Si possesses a valence state of −4, with a corresponding 2*p*_3/2_ binding energy of 97.5 eV [43], in excellent agreement with the value obtained in the present study. Considering electronegativity differences, Mg (1.23) and Si (1.74) yield a difference of 0.51, whereas in Nd_2_ScSi_2_, the average electronegativity of Nd (1.07) and Sc (1.20) is 1.135, resulting in a larger difference of 0.605 relative to Si [44, 45]. This suggests Nd and Sc atoms have a stronger electron‐donating capability toward Si than Mg in Mg_2_Si. Accordingly, the valence state of Si in Nd_2_ScSi_2_ can reasonably be assigned as −4.

Assuming Nd and Sc both adopt the +3 oxidation state while Si is −4, the overall charge balance of (+3 × 2) + (+3 × 1) + (−4 × 2) = +1 implies the presence of excess electrons that cannot be accommodated within the Si framework. These excess electrons are expected to localize within the structural voids, a hallmark feature of electrides. Indeed, in Nd_2_ScSi_2_, the anionic electrons exhibit partial delocalization through orbital hybridization with adjacent Nd and Sc atoms (Figure 1c). This delocalization not only accounts for the downward shifts in the binding energies of Nd (Figure 2e) and Sc (Figure S5) but also plays a critical role in enhancing the chemical robustness of Nd_2_ScSi_2_ compared with conventional electrides such as C12A7:e^−^ and Ca_2_N. Furthermore, the measured work function of Nd_2_ScSi_2_ (3.6 eV) is higher than that of typical electrides (below 3 eV), yet it remains superior to that of non‐electrides, indicating a moderate trade‐off between electron‐donating ability and chemical stability [12, 46]. Such a balanced electronic structure endows Nd_2_ScSi_2_ with both high reactivity and enhanced durability, rendering it a promising candidate for stable and efficient electrochemical applications.

### 2.3 Synthesis and Characterization of the Ru/Nd_2_ScSi_2_ Catalyst

**FIGURE 3:**
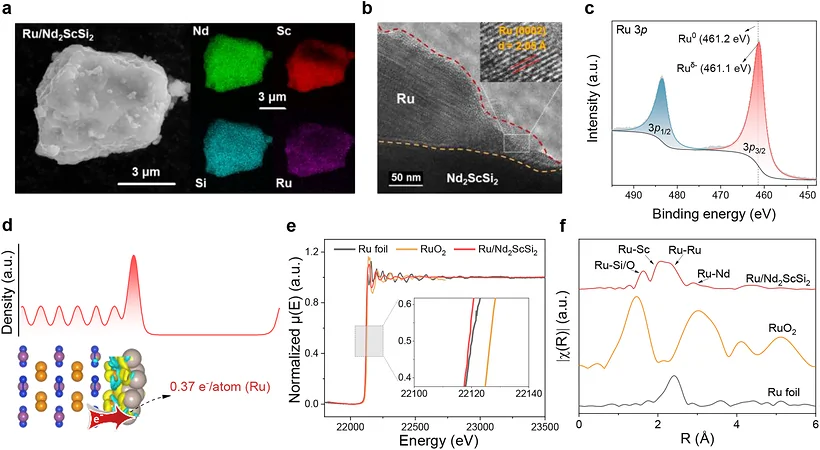
Characterization of the Ru/Nd_2_ScSi_2_ catalysts. (a) SEM image and EDX elemental mapping showing the distribution of elements in the Ru/Nd_2_ScSi_2_. (b) TEM image of Ru/Nd_2_ScSi_2_ and corresponding lattice fringe analysis. (c) XPS spectra of Ru species on the catalyst surface. (d) Charge distribution of Ru/Nd_2_ScSi_2_. Inset: differential charge density (cyan: charge depletion; yellow: charge accumulation). (e) Ru K‐edge XANES spectra. (f) Ru K‐edge EXAFS analysis showing Ru–Ru, Ru–Nd, and possible Ru–Sc or Ru–Si coordination. As Ru is present at a low concentration (5.3 wt.%) in the catalyst, the Ru/Nd_2_ScSi_2_ signal was amplified fourfold to enhance visibility.

In this work, the Ru/Nd_2_ScSi_2_ catalyst was synthesized via CVD of Ru. By employing Ru exclusively as a surface‐deposited active metal, this approach not only reduces the reliance on expensive noble metals such as Pt but also minimizes the overall noble metal loading. Following Ru deposition, the overall morphology of the Nd_2_ScSi_2_ support remains unchanged, and Ru is uniformly distributed across the surface, as confirmed by SEM and EDX elemental mapping (Figure 3a). To further elucidate the spatial distribution and structural characteristics of Ru, TEM analysis was conducted (Figure 3b). The images clearly reveal Ru nanoparticles anchored on the surface of Nd_2_ScSi_2_, with distinct and coherent interfaces between the Ru and the support. The measured lattice spacing of ∼0.205 nm matches well with the (0002) plane of hexagonal close‐packed Ru [31], indicating the high crystallinity of the deposited metal phase. Importantly, the intimate contact at the metal‐support interface may facilitate strong electronic interactions between the Ru nanoparticles and the electron‐rich Nd_2_ScSi_2_ electride support. Such interactions can modulate the *d*‐band structure of Ru, optimize the adsorption energies of key reaction intermediates, and thus enhance the catalytic performance [47, 48]. Therefore, both the structural characteristics and the electronic nature of the Ru/Nd_2_ScSi_2_ interface are expected to play a crucial role in governing the catalytic performance.

The anchoring of Ru atoms on the catalyst surface facilitates electron transfer from the substrate to Ru, driven by the work function difference between Nd_2_ScSi_2_ (3.6 eV) and Ru (4.7 eV) [49]. Owing to the metallic nature of both components, the interface is free from the Schottky barrier, thereby allowing seamless electron transport from the electride to the Ru active sites [50]. This prediction is corroborated by the XPS analysis of Ru (Figure 3c), in which the binding energy of Ru 3*p*_3/2_ is 461.1 eV, slightly lower than that of metallic Ru (461.2 eV) [51], indicating the presence of negatively charged Ru species. To further interrogate this interfacial electronic redistribution, charge density difference calculations were performed based on a Ru/Nd_2_ScSi_2_ slab model. As illustrated in Figure 3d, the plane‐averaged charge density difference along the *z*‐direction clearly indicates electron transfer from Nd_2_ScSi_2_ to the supported Ru atoms. This conclusion is quantitatively reinforced by Bader charge analysis, which reveals that each Ru atom acquires approximately 0.37 e^−^ on average. Collectively, these theoretical results exhibit strong agreement with the experimental observations, thereby confirming the substrate‐to‐Ru electron donation mechanism.

To gain a more comprehensive understanding of the local electronic and structural states of the supported Ru species, Ru K‐edge X‐ray Absorption Fine Structure measurements were conducted. First, the Ru K‐edge X‐ray absorption near‐edge structure (XANES) region was analyzed (Figure 3e). The XANES spectrum is consistent with the XPS analysis, confirming that Ru in Ru/Nd_2_ScSi_2_ is in a slightly negatively charged state relative to metallic Ru. To probe the local coordination environment, the extended X‐ray absorption fine structure (EXAFS) region was further analyzed. As shown in Figure 3f, besides the characteristic Ru─Ru bond, an additional longer Ru─Nd coordination is detected. Moreover, two slightly shorter bond lengths than that of the typical Ru─Ru distance are observed, which can be reasonably attributed to Ru─Sc or Ru─Si/O coordination. The corresponding wavelet transform analysis is provided in Figure S6 to further distinguish these scattering paths. These results collectively reveal a unique local coordination environment at the Ru/Nd_2_ScSi_2_ interface, highlighting strong electronic and structural interactions between the supported Ru species and the substrate.

### 2.4 Alkaline HER Performance

**FIGURE 4:**
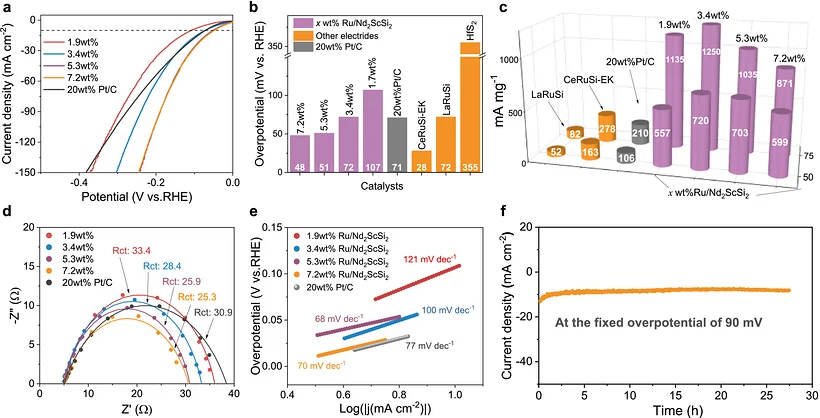
Electrocatalytic HER performance of Ru/Nd_2_ScSi_2_ catalysts. (a) iR‐corrected linear sweep voltammetry (LSV) curves. The horizontal dashed line indicates a current density of −10 mA cm^−2^. The *x* wt.% in the labels represents the content of Ru in Ru/Nd_2_ScSi_2_. (b) Comparison of overpotentials at −10 mA cm^−2^ for Ru/Nd_2_ScSi_2_, 20 wt.% Pt/C, and other reported electride‐based catalysts [37, 38, 52]. (c) Mass activity comparison of the studied and previously reported catalysts [37, 38], where 3.4 wt.% Ru/ Nd_2_ScSi_2_ exhibits the best performance. (d, e) Electrochemical impedance spectra (EIS) and Tafel slopes of Ru/Nd_2_ScSi_2_ catalysts with different Ru loadings and 20 wt.% Pt/C. (f) Chronoamperometry test at the fixed overpotential of 90 mV for up to 27.5 h.

To assess the electrocatalytic performance of Ru‐supported Nd_2_ScSi_2_ (Ru/Nd_2_ScSi_2_) catalysts toward alkaline HER, linear sweep voltammetry (LSV) measurements were carried out at different Ru loadings. As shown in Figure 4a, the HER activity increases progressively with increasing Ru content. However, the improvement becomes negligible beyond a loading of 5.3 wt.%, as the catalyst containing 7.2 wt.% Ru exhibits nearly identical performance to the 5.3 wt.% sample. The electrochemically active surface area (ECSA) results (Figures S7 and S8) also demonstrate a clear trend with increasing Ru loading: 1.9 wt.% (0.243 mF cm^−2^), 3.4 wt.% (0.325 mF cm^−2^), 5.3 wt.% (0.855 mF cm^−2^), and 7.2 wt.% (0.865 mF cm^−2^). The progressive increase in double‐layer capacitance (C_dl_) correlates well with the enhanced HER activity observed in terms of overpotential and current density. Notably, the increase from 5.3 wt.% to 7.2 wt.% is marginal (only about 1.2%), which aligns with the saturation trend in HER performance. This plateau behavior can be attributed to the intrinsically low specific surface area of the Nd_2_ScSi_2_ support (2.3 m^2^ g^−1^), which limits the dispersion of additional Ru atoms. Consequently, excess Ru tends to aggregate rather than generate new active sites, thereby failing to further enhance the catalytic activity.

A direct comparison of overpotentials at a current density of −10 mA cm^−2^ is presented in Figure 4b. The catalyst with 3.4 wt.% Ru achieves an overpotential of 72 mV, nearly identical to that of commercial 20 wt.% Pt/C (71 mV). Further increasing the Ru loading to 5.3 wt.% and 7.2 wt.% reduces the overpotential to 51 mV and 48 mV, respectively, thereby surpassing the catalytic activity of 20 wt.% Pt/C. Although these values are slightly higher than those of the electride‐based CeRuSi‐EK catalyst (28 mV, prepared via ethylenediaminetetraacetic acid and KOH etching) [38], Ru/Nd_2_ScSi_2_ requires substantially less Ru metal, offering clear advantages in terms of cost‐effectiveness and scalability.

To evaluate the efficiency of noble metal utilization, the mass activities of Ru/Nd_2_ScSi_2_ and Pt/C catalysts were calculated at overpotentials of 50 and 75 mV (Figure 4c). Remarkably, the 3.4 wt.% Ru/Nd_2_ScSi_2_ sample exhibited the highest mass activity, delivering 6.8 and 6.0 times that of 20 wt.% Pt/C at 50 and 75 mV, respectively. All Ru/Nd_2_ScSi_2_ catalysts also displayed significantly higher mass activity than CeRuSi‐EK, with improvements of 3.4–4.4 times at 50 mV and 3.1–4.5 times at 75 mV, further validating the superior efficiency of Ru/Nd_2_ScSi_2_. Collectively, these findings indicate that a Ru loading of approximately 3 wt.%–5 wt.% represents the optimal balance between active site utilization and catalyst efficiency. Importantly, this rational loading not only maintains high intrinsic HER activity but also maximizes mass‐normalized performance through efficient utilization of the active metal. Moreover, the superior activity at this optimal loading reveals that interfacial electronic interactions between Ru and Nd_2_ScSi_2_, rather than Ru content alone, play a decisive role in determining the catalytic performance. This conclusion is well supported by the XANES and EXAFS results, which revealed distinctive Ru─Nd and Ru─Sc/Si coordinations, thereby establishing a direct correlation between the interfacial structure and the observed catalytic enhancement.

Electrochemical impedance spectroscopy (EIS) was employed to probe the charge‐transfer kinetics of the Ru/Nd_2_ScSi_2_ catalysts. As shown in Figure 4d, all Ru/Nd_2_ScSi_2_ samples exhibit significantly lower charge‐transfer resistance (*R*_ct_) compared with Pt/C, indicative of accelerated electron transport during the HER process [53]. The progressive decrease in *R*_ct_ with increasing Ru loading reflects enhanced interfacial conductivity and a greater number of accessible active sites. However, further increasing the Ru content from 5.3 wt.% to 7.2 wt.% yields only a marginal reduction in *R*_ct_, again suggesting saturation of surface active sites due to the limited specific surface area of Nd_2_ScSi_2_.

The HER kinetics were further examined via Tafel analysis (Figure 4e). With increasing Ru loading, the availability of more active sites for water activation results in a decrease in the Tafel slope from 121 to 68 mV dec^−1^. The large initial slope (121 mV dec^−1^) at low Ru loading suggests that water dissociation (Volmer step: H_2_O + e^−^ → H + OH^−^) is the rate‐limiting process. As Ru content increases, simultaneous activation of additional water molecules becomes possible, shifting the rate‐determining step toward the Heyrovsky (H + H_2_O + e^−^ → H_2_↑ + OH^−^) and Tafel (2H* → H_2_↑) processes [54]. The slope stabilizes at ∼70 mV dec^−1^ when the Ru loading reaches 5.3 wt.%, signifying that this is the optimal Ru content for water activation. Notably, this value is even lower than that of commercial Pt/C (77 mV dec^−1^), highlighting the superior kinetic properties of Ru/Nd_2_ScSi_2_.

The long‐term catalytic durability of Ru/Nd_2_ScSi_2_ was further assessed by chronoamperometry at a fixed overpotential of 90 mV. As shown in Figure 4f, the catalyst sustains a stable current density over 27.5 h with minimal decay, underscoring its robust stability. Post‐stability structural characterizations reinforce this observation: XRD patterns (Figure S9) reveal no detectable phase changes, SEM images (Figure S10) confirm a smooth and intact catalyst surface, and EDX analysis shows that the Nd:Sc:Si atomic ratio remains close to the original stoichiometry (2.1:1:1.9). TEM images confirm that Ru nanoparticles maintain their original morphology without obvious aggregation (Figure S11). Inductively coupled plasma atomic emission spectroscopy (ICP‐AES) analysis shows that the Ru loading changes from 5.3 wt.% to 5.0 wt.%, within normal measurement uncertainty, indicating negligible Ru dissolution. However, XPS analysis reveals a slight positive shift in Ru binding energy after testing (Figure S12), suggesting minor surface oxidation of Ru under the electrochemical environment. This partial oxidation is likely responsible for the slight activity decay. These results collectively demonstrate excellent structural and compositional integrity of the catalyst under prolonged operation. Crucially, the durability can be ascribed to the robust interfacial coordination environment of Ru with Nd, Sc, and Si atoms, as revealed by XANES and EXAFS analyses, which stabilizes the active sites against aggregation or structural degradation during extended HER operation.

### 2.5 Mechanism Investigation

**FIGURE 5:**
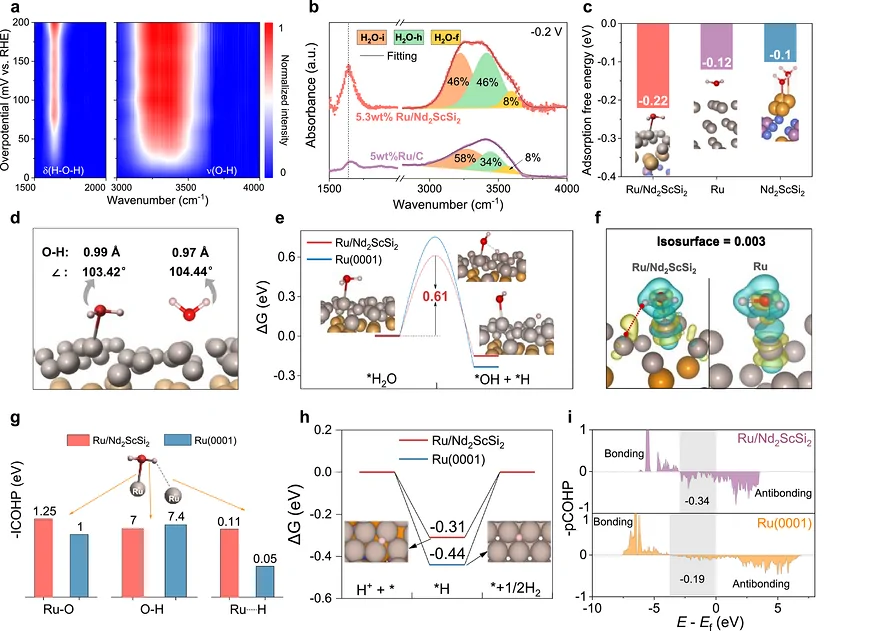
Mechanistic studies of HER on Ru/Nd_2_ScSi_2_. (a) In situ infrared spectroscopy (IR) of Ru/Nd_2_ScSi_2_ collected from 0 to 200 mV. (b) Operando IR spectra of Ru/Nd_2_ScSi_2_ and Ru/C at −0.2 V. (c) Comparison of water adsorption free energy on Ru/Nd_2_ScSi_2_, Ru(0001), and Nd_2_ScSi_2_. (d) Structural evolution of H_2_O adsorption configurations on Ru/Nd_2_ScSi_2_. (e) Transition state comparison of H_2_O dissociation on Ru/Nd_2_ScSi_2_ and Ru(0001) surfaces. (f) Differential charge density plots of H_2_O adsorbed on Ru/Nd_2_ScSi_2_ and Ru(0001). (g) Negative Integrated Crystal Orbital Hamilton Population comparison of key bonds (Ru─O, O─H, Ru─H) formed upon water activation. (h) Free energy profiles for hydrogen adsorption on Ru/Nd_2_ScSi_2_ and Ru(0001). (i) Comparison of −pCOHP for Ru─H bonds for bridge‐site adsorption on Ru/Nd_2_ScSi_2_ and for hollow‐site adsorption on Ru(0001).

To elucidate the HER mechanism of Ru/Nd_2_ScSi_2_ under alkaline conditions, we conducted a comprehensive study integrating in situ infrared spectroscopy (IR) and DFT simulations. As shown in Figure 5a, the in situ IR of the 5.3 wt.% Ru/Nd_2_ScSi_2_ under applied potentials from 0 to 200 mV exhibit two characteristic absorption regions: one centered around 1650 cm^−1^, corresponding to the δ(H─O─H) bending vibration of H_2_O, and a broad band spanning 3000–3700 cm^−1^, ascribed to the ν(O─H) stretching vibrations of interfacial water species [55, 56]. As the potential increases, both bands show clear intensity enhancement and spectral broadening, indicating increased amounts and stronger adsorption of water or hydroxyl species at the catalyst interface, in accordance with the electrochemical Stark effect.

Further insights are obtained from operando infrared spectra at −0.2 V, as shown in Figure 5b, where 5.3 wt.% Ru/Nd_2_ScSi_2_ is compared with 5.0 wt.% Ru/C and pristine Nd_2_ScSi_2_. The bending vibration band appears at a lower wavenumber in 5.3 wt.% Ru/Nd_2_ScSi_2_ (1640 cm^−1^) than in 5.0 wt.% Ru/C (1659 cm^−1^), indicating stronger polarization of water molecules bound to the catalyst surface [54]. The significantly larger area of this band further suggests a higher density of adsorbed water on 5.3 wt.% Ru/Nd_2_ScSi_2_. In the O─H stretching region (3000–3700 cm^−1^), deconvolution reveals contributions from three types of interfacial water: free water (H_2_O–f), cation‐hydrated water (H_2_O–h), and strongly hydrogen‐bonded ice‐like water (H_2_O–i) [57]. Among these, H_2_O–f and H_2_O–h both are known to serve as more reactive hydrogen sources due to their weaker hydrogen bonding and greater mobility [6, 56, 57]. Compared with Ru/C, Ru/Nd_2_ScSi_2_ not only exhibits a total O─H absorption band area 3 times greater, but also shows a notable increase in the proportion of these reactive species from 42% to 54% (see Table S1). These results suggest both an increased number and improved distribution of catalytically favorable water adsorption modes on Ru/Nd_2_ScSi_2_.

Figure 5c presents the calculated adsorption free energies of H_2_O on Ru/Nd_2_ScSi_2_, Ru(0001), and Nd_2_ScSi_2_, with model construction details provided in Figures S13–S16. Among these systems, Ru/Nd_2_ScSi_2_ exhibits the most negative adsorption free energy, signifying the strongest interaction with H_2_O. By contrast, Ru(0001) shows a weaker and comparable adsorption strength, consistent with the infrared spectra, whereas Nd_2_ScSi_2_ displays the weakest water‐binding ability and poor catalytic activity (Figure S17). These results clearly indicate that Ru serves as the effective active site in the Ru/Nd_2_ScSi_2_ system. Structural analysis (Figure 5d) reveals that upon H_2_O adsorption on Ru/Nd_2_ScSi_2_, the O─H bond lengthens while the H─O─H bond angle decreases, indicative of enhanced polarization and pre‐activation of the water molecule. This conclusion is further supported by the calculated water dissociation energy barrier (Figure 5e; Figure S18), which is significantly lower on Ru/Nd_2_ScSi_2_ (0.61 eV) compared with Ru(0001) (0.76 eV).

Differential charge density maps (Figure 5f) further show pronounced charge redistribution upon H_2_O adsorption on Ru/Nd_2_ScSi_2_ at identical isosurface values, whereas Bader charge analysis suggests negligible net charge transfer. Importantly, localized electronic interactions emerge between the H atom (Bader charge +1) and the neighboring negatively charged Ru atom (Bader charge −0.44), attributed to Coulombic attraction. This interaction is corroborated by the differential electrostatic potential maps (Figure S19), which reveal enhanced Ru─H interaction. To further quantify bonding characteristics during the dissociation process, −ICOHP values were evaluated (Figure 5g). Ru/Nd_2_ScSi_2_ shows stronger Ru─O bonding and weaker O─H bonding compared to Ru(0001), implying more efficient H_2_O activation and easier O─H bond cleavage. Notably, Ru─H bonding is significantly strengthened on Ru/Nd_2_ScSi_2_ (0.11 eV) relative to Ru(0001) (0.05 eV), highlighting the critical role of electride‐induced negatively charged Ru in promoting O─H bond scission via reinforced Ru‐H electrostatic interactions.

The hydrogen adsorption behavior was further investigated (Figure 5h). The calculated Δ*G*(H*) on Ru/Nd_2_ScSi_2_ (−0.31 eV) is closer to the thermoneutral value (Δ*G* = 0 eV) than that on Ru(0001) (−0.44 eV), indicating more favorable adsorption of hydrogen intermediates for HER. Interestingly, while hydrogen preferentially adsorbs at hollow sites on Ru(0001) (Figure S20), in agreement with previous studies [58], it spontaneously migrates to bridge sites on Ru/Nd_2_ScSi_2_ (Figure S21). This unusual behavior can be ascribed to the enhanced surface electron density of Ru atoms in the Ru/Nd_2_ScSi_2_ system, as confirmed by Bader charge analysis, which reveals an average electron gain of 0.37 e^−^ per Ru atom (Figure 3d).

Charge density difference (Figure S22) and Bader charge analysis (Figure S23) consistently show that both Ru and H atoms carry negative charges. In the hollow configuration, H simultaneously coordinates with three negatively charged Ru atoms, generating a highly localized electron density. This configuration leads to strong Coulomb repulsion between Ru^δ−^ and H^−^ species, thereby weakening and destabilizing the Ru─H bonding. It is therefore reasonable to infer that the excessive electron density at hollow sites results in antibonding orbital occupation between Ru^δ−^ and H^−^, which destabilizes hydrogen adsorption at highly coordinated sites. In contrast, bridge sites, characterized by lower coordination and a more moderate electron density, provide a more stable adsorption configuration.

Moreover, the negative charge on Ru^δ−^ increases the *d*‐electron population, leading to a contraction of the Ru *d*‐band (Figure S24). COHP analysis (Figure 5i) shows that this *d*‐band contraction shifts the antibonding states downward in energy, causing them to become partially occupied below the Fermi level. As a result, the integrated antibonding value changes from −0.19 for hollow‐site adsorption on Ru(0001) to −0.34 for bridge‐site adsorption on Ru/Nd_2_ScSi_2_, reflecting an enhanced antibonding interaction that weakens the Ru─H bond. Compared with pristine Ru, the reduced coordination at the bridge site thus weakens H adsorption, thereby generating more favorable conditions for hydrogen evolution.

**FIGURE 6:**
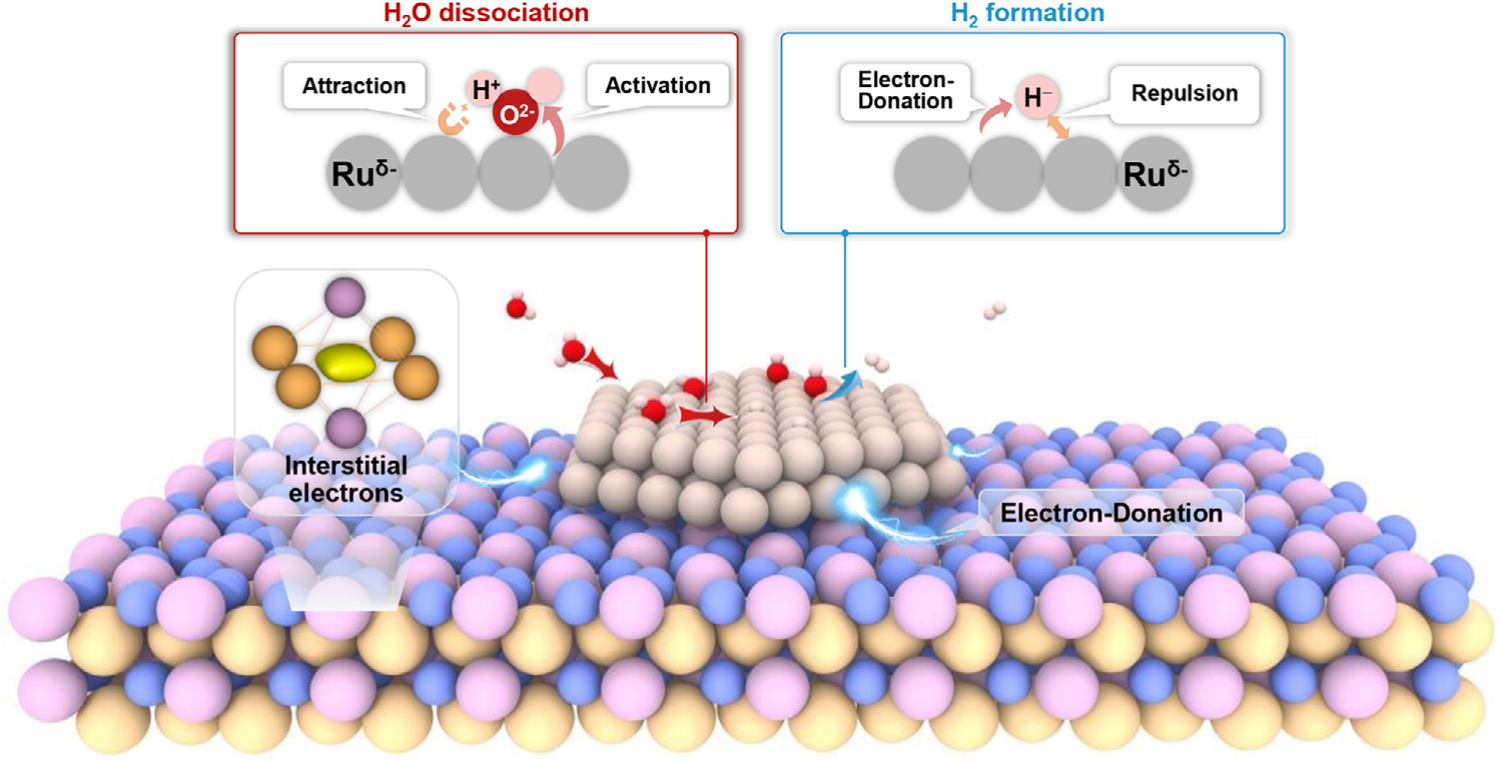
Schematic illustration of the dual catalytic mechanisms on Ru/Nd_2_ScSi_2_: the electride‐induced negative‐valence Ru promotes water adsorption/activation by enhancing H_2_O polarization while simultaneously facilitating H desorption by Coulombic repulsion, thereby boosting HER activity.

In summary, the Ru/Nd_2_ScSi_2_ catalyst exhibits exceptional HER activity in alkaline media, which can be ascribed to the unique negatively charged state of Ru induced by the strongly electron‐donating electride support. This electronic modulation not only promotes H_2_O adsorption and activation but also optimizes hydrogen desorption, thereby accelerating the overall H_2_ evolution process. As schematically illustrated in Figure 6, these findings underscore the critical role of electronic structure engineering in dictating catalytic pathways. More importantly, this work establishes a generalizable strategy for the rational design of electride‐based electrochemical catalysts, in which the pronounced interfacial charge transfer and the consequent electronic band modulation of supported active sites can be deliberately engineered to overcome intrinsic kinetic barriers in the HER and, potentially, in other pivotal electrochemical processes. Looking forward, this strategy can be further extended through high‐throughput screening of water‐stable electrides, combined with experimental validation and interfacial engineering, to establish a predictive framework linking electronic structure to catalytic performance.

## 3 Conclusion

In this work, we demonstrate for the first time a supported electride‐based catalyst for alkaline hydrogen evolution, Ru/Nd_2_ScSi_2_, in which the electride functions as an electron‐rich support to modulate the catalytic properties of Ru active sites. The catalyst delivers exceptional HER activity in 1.0 m KOH, requiring an overpotential as low as 48 mV at −10 mA cm^−2^, surpassing the performance of commercial Pt/C and many state‐of‐the‐art Ru‐based systems. Mechanistic insights obtained from in situ IR and DFT calculations consistently highlight the central role of electride‐induced negatively charged Ru centers. These electron‐enriched sites simultaneously promote water adsorption and dissociation, lower the kinetic barrier for O─H bond cleavage, and optimize hydrogen intermediate binding energies through tailored electronic interactions at the metal‐electride interface. The synergy between Ru and the electride support results in both high intrinsic activity and efficient Ru utilization, thereby reconciling catalytic performance with cost‐effectiveness. Beyond demonstrating outstanding catalytic performance, this study establishes the integration of electrides with high chemical stability into supported architectures as a powerful platform for electronic structure engineering. By rationally tuning surface charge states through support‐metal interactions, this approach opens a generalizable strategy for the design of efficient, durable, and economically viable HER electrocatalysts in alkaline environments.

## 4 Methods

### 4.1 Catalyst Synthesis

All metals used in this study were purchased from Aladdin Reagent Co., Ltd. The Nd_2_ScSi_2_ compound was synthesized via a one‐step arc melting method. High‐purity Nd (99.9%), Sc (99.9%), and Si (99.9%) pieces were mixed in a molar ratio of 2:1:2 and arc‐melted five times to ensure compositional homogeneity. The resulting ingot was annealed at 1000°C for two weeks to enhance phase purity, then mechanically crushed and ground in an argon‐filled glovebox before further use. The Ru/Nd_2_ScSi_2_ catalyst was prepared using a chemical vapor deposition (CVD) technique. A predetermined amount of Ru_3_(CO)_12_ was mixed with 0.5 g of the synthesized electride in an argon‐filled glovebox. The mixture was sealed in a quartz tube and subjected to the following heating program: ramped to 70°C at 1°C min^−1^ and held for 1 h; further heated to 120°C over 2 h and held for 1 h; then increased to 250°C over 2.5 h and maintained for 2 h; finally cooled down to room temperature naturally. The resulting catalyst was used directly for performance evaluation and characterization without any additional treatment.

### 4.2 Sample Characterizations

The crystal structures of the samples were characterized by powder X‐ray diffraction (XRD) using a Bruker D8 DISCOVER A25 diffractometer equipped with a Cu Kα radiation source. The morphologies and microstructures were examined using a Hitachi S‐4800 field‐emission SEM, and the elemental distributions were obtained via EDX mapping. Further structural analysis at the nanoscale was conducted using a Titan Themis Z G3 Cs‐corrected TEM.

The surface electronic properties were analyzed using UPS and XPS. UPS measurements were performed on a Thermo Scientific ESCALAB 250Xi system equipped with a VGS Class150 electron analyzer and a discharge lamp emitting He‐I radiation (21.2 eV). XPS spectra were acquired using a Thermo Scientific K‐Alpha system with a monochromatic Al Kα X‐ray source. All binding energies were calibrated against the C 1*s* peak at 284.8 eV. EXAFS data for the synthesized catalysts were measured in transmission mode using Oxford ionization detectors at the Pohang Accelerator Laboratory. Room‐temperature Ru K‐edge XAS spectra were collected specifically on beamline 6D at PAL. Standard background subtraction, normalization, and Fourier transformation procedures were applied using the ATHENA program [59]. The Ru content was quantified by ICP‐ AES using an Agilent 5110 instrument. The specific surface area of the samples was determined by the Brunauer–Emmett–Teller (BET) method, based on niAtrogen adsorption–desorption isotherms measured at −196°C using an ASAP 2020 Plus automatic gas adsorption analyzer.

H_2_‐TPD was performed on a PCA‐2200 to investigate the hydrogen desorption behavior of the materials. Prior to the TPD measurement, the Nd_2_ScSi_2_ powders were first pre‐treated in a high‐purity Ar flow (30 mL min^−1^) at 300°C to clean the sample surface, and then cooled to 200°C. Subsequently, hydrogenation was carried out at 200°C by exposing the samples to a mixed gas flow of pure H_2_ for 1 h. After hydrogenation, the samples were purged with pure Ar at 200°C for 1 h to remove any physisorbed hydrogen, followed by cooling to 50°C. The TPD profile was then obtained by heating the samples from 50°C to 800°C at a ramp rate of 10°C min^−1^ in an Ar flow, while the signal of desorbed H_2_ was continuously recorded by a thermal conductivity detector.

### 4.3 Electrochemical Measurements

Electrochemical measurements were conducted at room temperature using a typical three‐electrode system, connected to an Autolab (MULTI AUTOLAB M204, Switzerland). Pt served as the counter electrode, while Ag/AgCl acted as the reference electrode, in a 1.0 m KOH electrolyte. For the working electrodes, 10 mg of catalyst powder was dispersed in a mixture of ethanol and ultrapure water with 50 µL Nafion solution (5 wt.%), which underwent ultrasonication to achieve a homogeneous ink. Subsequently, 10 µL of ink was cast onto the glassy carbon electrode and allowed to dry naturally.

Polarization curves were generated by scanning potential at a rate of 5 mV s^−1^. These curves were then plotted to depict the relationship between overpotential (η) and log current (log j) to derive Tafel plots for assessing HER kinetics. EIS data of electrocatalysts were gathered with an amplitude of 5 mV over a frequency range from 10 000 to 0.01 Hz, maintaining an applied overpotential of −0.09 V. The electrochemical double‐layer capacitance (C_dl_) of Ru/Nd_2_ScSi_2_ catalysts with varying Ru loadings using cyclic voltammetry in the non‐Faradaic region (0.37–0.53 V vs. RHE) at scan rates of 20 to 100 mV s^−1^. The C_dl_ values were derived from the linear relationship between the current density difference and the scan rate. The measured potentials were converted against a reversible hydrogen electrode (RHE) according to the following equation:

E(RHE)=E(Hg/HgO)+0.098+0.0592pH.

### 4.4 Potential‐Dependent In Situ ATR‐FTIR Spectroscopy

In situ ATR‐FTIR spectroscopy was performed using a Bruker FTIR spectrometer equipped with a silicon prism. Before each measurement, a thin gold film was chemically deposited onto the prism surface. Powdered catalysts were dispersed in ethanol and sonicated for over 30 min. Subsequently, 20 µL of the resulting ink was deposited onto the gold film to form the working electrode. The working electrode was maintained at each potential for 5 min. The Pt sheet and Hg/HgO electrode were used as the counter electrode and the reference electrode, respectively. The measurement was conducted in the 1.0 m KOH electrolyte.

### 4.5 Density Functional Theory Calculations

All first‐principles calculations in this study were carried out using the Vienna Ab Initio Simulation Package (VASP) [60], based on DFT and the projector‐augmented wave method [61]. The electron exchange‐correlation interactions were treated using the generalized gradient approximation in the Perdew‐Burke‐Ernzerhof formulation [62]. For structural relaxations, plane‐wave cutoff energy of 520 eV and k‐point mesh density of 2π × 0.03 Å^−1^ were employed. The atomic positions and unit cell volumes were fully optimized until the residual forces on all atoms were less than |0.05| eV Å^−1^ and the total energy change was below 10^−6^ eV. All calculations considered spin polarization. In accordance with the experimental XPS results, the Nd_3 pseudopotential was used during the simulations, with on‐site Coulomb interactions (U) applied to the *f*‐electrons, following values reported in the literature [63]. These settings were consistently applied in all subsequent electronic structure calculations. For the calculations of band structures, DOS, and ELF, a denser k‐point mesh of 2π × 0.02 Å^−1^ was utilized to ensure higher accuracy. In surface model calculations, a supercell was constructed with a vacuum layer of 20 Å along the c‐axis to eliminate spurious interactions due to periodic boundary conditions. Additionally, van der Waals interactions were corrected using the semi‐empirical DFT‐D3 method proposed by Grimme [64]. The transition states were initially located using the climbing image nudged elastic band method and subsequently refined using the Dimer method for improved accuracy [65, 66]. Atomic charges were analyzed using the Bader charge decomposition based on the charge density distributions [67]. The charge density difference and deformation electron potential were calculated to analyze electron transfer and interatomic interactions, respectively. The crystal and electronic structure visualizations in this work were performed using VESTA and OVITO [68, 69].

The change in Gibbs free energy (ΔG) for reaction intermediates was calculated using the following equation:

ΔG=ΔE+ΔZPE−TΔS

where ΔE is the reaction energy calculated using DFT; ΔZPE is the zero‐point energy obtained through vibrational analysis using the VASPKIT code [70]; T is the temperature that is set to 300 K in this work, and ΔS is the change in entropy.

## Funding

The National Key Research and Development Program of intergovernmental cooperation in science and technology (Grant No. 2022YFE0141100). The Fundamental Research Funds for the Central Universities (Grant No. D5000240207). JST Mirai Project (JPMJMI21E9).

## Conflicts of Interest

The authors declare no conflicts of interest.

## Supporting information




**Supporting File**: advs74939‐sup‐0001‐SuppMat.docx.

## Data Availability

The data that support the findings of this study are available from the corresponding author upon reasonable request.
