# Supplementary material for: Electride‐Induced Electronic Modulation of Ruthenium Catalyst for Highly Efficient Alkaline Hydrogen Evolution
Source: Adv Sci (Weinh). 2026 Mar 24;13(31):e74939. doi: 10.1002/advs.74939 (PMC13587976; doi:10.1002/advs.74939)
Supplement: Supplementary file 1 [file ADVS-13-e74939-s001.docx]

Supporting Information

**Electride-Induced Electronic Modulation of Ruthenium Catalyst for Highly Efficient Alkaline Hydrogen Evolution**

*Zhiqi Wang, Jianan Su, Cheng Li, Yutong Gong, Hideo Hosono*, Junjie Wang**

Zhiqi Wang, Jianan Su, Cheng Li, Yutong Gong, Junjie Wang

State Key Laboratory of Solidification Processing, School of Materials Science and Engineering, Northwestern Polytechnical University, Xi'an, Shaanxi 710072, People's Republic of China.

Xi'an Key Laboratory of Intelligent Design of Energy Catalytic Materials, Northwestern Polytechnical University, Xi'an, Shaanxi 710072, People's Republic of China.

E-mail: wang.junjie@nwpu.edu.cn

Hideo Hosono

MDX Research Center for Element Strategy, Institute of Integrated Research, Institute of Science Tokyo, 4259 Nagatsuta, Midori-ku, Yokohama, 226-8503 Japan

E-mail: hosono.h.aa@m.titech.ac.jp

Zhiqi Wang and Jianan Su contributed equally to this work.

**ORCID**

Zhiqi Wang ID: 0009-0001-5951-1230

Jianan Su ID: 0000-0002-2849-4562

Cheng Li ID: 0000-0003-4069-5989

Yutong Gong ID: 0000-0003-4676-9547

Hideo Hosono ID: 0000-0001-9260-6728

Junjie Wang ID: 0000-0002-6428-2233

Figure S1. Energy profile of hydrogen desorption from Nd_2_ScSi_2_ at 0 K, obtained from DFT calculations.

Figure S2. Calculated energy profile for hydrogen diffusion along the [001] direction in Nd_2_ScSi_2_ using the CI-NEB method.

Figure S3. TEM images of Nd_2_ScSi_2_.

Figure S4. High-resolution XPS spectrum of the C 1*s* region.

Figure S5. X-ray photoelectron spectroscopy (XPS) analysis of Sc in Nd_2_ScSi_2._

Figure S6. Wavelet-transform (WT) plot of (a) Ru powder, (b) RuO_2_, (c) Ru/Nd_2_ScSi_2_.

Figure S7. Cyclic voltammetry curves in the non-Faradaic region (0.37–0.53 V vs. RHE) for Ru/Nd_2_ScSi_2_ with different Ru loadings: (a) 1.9 wt%, (b) 3.4 wt%, (c) 5.3 wt%, and (d) 7.2 wt%.

Figure S8. Electrochemical double-layer capacitance measurements for Ru/Nd_2_ScSi_2_ with different Ru loadings: (a) 1.9 wt%, (b) 3.4 wt%, (c) 5.3 wt%, and (d) 7.2 wt%.

Figure S9. XRD patterns of Ru/Nd_2_ScSi_2_ before and after the stability test.

Figure S10. SEM image and EDX analysis of Ru/Nd_2_ScSi_2_ after stability testing.

Figure S11. TEM characterization of Ru/Nd_2_ScSi_2_ before (a–c) and after (d–f) the stability test. (a, d) HAADF-STEM images with corresponding EDS elemental mapping; (b, e) high-magnification TEM images of Ru nanoparticles; (c, f) selected area electron diffraction (SAED) patterns of Ru.

Figure S12. XPS spectra of Ru 3p for Ru/Nd_2_ScSi_2_ before and after the stability test. The Ru 3p_3/2_ binding energy shifts from 461.1 eV to 462.9 eV after testing, indicating partial oxidation of Ru during long-term electrochemical operation.

|  | H–i | | H–h | | H–f | |
| --- | --- | --- | --- | --- | --- | --- |
|  | Integrated area | Relative content | Integrated area | Relative content | Integrated area | Relative content |
| 5.3wt% Ru/Nd_2_ScSi_2_ | 4.81 | 46% | 4.82 | 46% | 0.82 | 8% |
| 5.0wt% Ru/C | 2.67 | 58% | 1.56 | 34% | 0.35 | 8% |

Table S1. Integrated areas and relative percentage contents of different interfacial H_2_O species (H_2_O–f, H_2_O–h, and H_2_O–i) for Ru/Nd_2_ScSi_2_ and Ru/C catalysts, derived from O-H stretching region (3000–3700 cm⁻¹) in the in situ IR spectra.

Figure S13. Structural model of Ru/Nd_2_ScSi_2_ showing side and top views.

Note: A monolayer of Ru atoms was constructed on the Nd_2_ScSi_2_(001) surface to represent the optimal loading scenario, as experimentally observed that excessive Ru loading beyond a threshold shows no further enhancement in HER activity. We acknowledge that the real system involves Ru nanoparticles rather than a continuous monolayer. However, this model is designed to capture the essential electronic interaction between Ru and the electride support, specifically the formation of Ru^δ⁻^ species through interfacial charge transfer, rather than to replicate the exact nanoparticle geometry. This simplified approach effectively describes the characteristics of negatively charged Ru in water dissociation and hydrogen adsorption processes, and its excellent agreement with experimental observations validates the computational approach.

Figure S14. Structural models showing side and top views of (a) Nd_2_ScSi_2_ and (b) Ru surfaces.

Note: The Nd_2_ScSi_2_(001) surface was selected based on TEM characterization results, which identified (001) as the preferentially exposed facet. The Ru(0001) surface was chosen as it represents the most commonly exposed surface of hexagonal close-packed Ru, providing a well-established platform for studying surface reactions.

Figure S15. Optimized configurations of six different water adsorption structures on the Ru/Nd_2_ScSi_2_, along with their corresponding adsorption energies (E_ads_).

Figure S16. Optimized water adsorption configurations on (a) Nd_2_ScSi_2_(001) and (b) Ru(0001) surfaces.

Figure S17. Linear sweep voltammetry (LSV) curves comparing the HER activity of pristine Nd_2_ScSi_2_ and Ru-loaded Nd_2_ScSi_2_.
Figure S18. Initial, transition, and final states of water dissociation process on the Ru(0001) surface.

Figure S19. Differential electrostatic potential analysis showing distinct charge redistribution at the Ru/Nd_2_ScSi_2_ interface.

Figure S20. Initial and optimized adsorption configurations of H on Ru(0001), including hollow, bridge, and on-top sites.

Figure S21. Initial and optimized adsorption configurations of H on Ru/Nd_2_ScSi_2_, including hollow and on-top sites.

Figure S22. Charge density difference for hydrogen adsorbed at the bridge site on negatively charged Ru. Yellow and blue isosurfaces represent electron accumulation and depletion, respectively.

Figure S23. Bader charge analysis for hydrogen adsorption on (a) Ru/Nd_2_ScSi_2_, comparing H on (b) Ru(0001).

Note: Both configurations show electron transfer from Ru to H. The results show that while adsorbed H gains electrons from nearby Ru atoms, these Ru atoms are replenished by electrons from more distant Ru sites, leading to an overall Bader charge close to zero.

Figure S24. Projected density of states (DOS) of Ru *d* orbitals for Ru(0001) and Ru/Nd_2_ScSi_2_.
